# Response Surface Analysis of Genomic Prediction Accuracy Values Using Quality Control Covariates in Soybean

**DOI:** 10.1177/1176934319831307

**Published:** 2019-03-07

**Authors:** Diego Jarquín, Reka Howard, George Graef, Aaron Lorenz

**Affiliations:** 1 Department of Agronomy and Horticulture, University of Nebraska–Lincoln, Lincoln, NE, USA; 2 Department of Statistics, University of Nebraska–Lincoln, Lincoln, NE, USA; 3 Department of Agronomy and Plant Genetics, University of Minnesota, Minneapolis, MN, USA

**Keywords:** genomic prediction, quality control, response surface, soybean, imputation, Random Forest, minor allele frequency, missing marker score

## Abstract

An important and broadly used tool for selection purposes and to increase yield and genetic gain in plant breeding programs is genomic prediction (GP). Genomic prediction is a technique where molecular marker information and phenotypic data are used to predict the phenotype (eg, yield) of individuals for which only marker data are available. Higher prediction accuracy can be achieved not only by using efficient models but also by using quality molecular marker and phenotypic data. The steps of a typical quality control (QC) of marker data include the elimination of markers with certain level of minor allele frequency (MAF) and missing marker values and the imputation of missing marker values. In this article, we evaluated how the prediction accuracy is influenced by the combination of 12 MAF values, 27 different percentages of missing marker values, and 2 imputation techniques (IT; naïve and Random Forest (RF)). We constructed a response surface of prediction accuracy values for the two ITs as a function of MAF and percentage of missing marker values using soybean data from the University of Nebraska–Lincoln Soybean Breeding Program. We found that both the genetic architecture of the trait and the IT affect the prediction accuracy implying that we have to be careful how we perform QC on the marker data. For the corresponding combinations MAF-percentage of missing values we observed that implementing the RF imputation increased the number of markers by 2 to 5 times than the simple naïve imputation method that is based on the mean allele dosage of the non-missing values at each loci. We conclude that there is not a unique strategy (combination of the QCs and imputation method) that outperforms the results of the others for all traits.

## Introduction

Soybean (Glycine max [L.] Merr.) is one of the most important crops grown worldwide, and it contributes significantly to food production. According to the World Agricultural Outlook Report by the United States Department of Agriculture (USDA), in 2016, the United States produced more than 100 million of tons of soybean, and in 2018, it was projected that the production would be close to 120 million of tons. Soybean provides a high-quality vegetable protein that is used primarily in animal feed and for human food uses and is the leading vegetable protein produced worldwide. Soybean oil is a high-quality vegetable oil that is used in food, feed, and industrial applications. Thus, with the increasing demand for high-quality and sustainable food production, it is necessary to improve soybean yield and increase the performance through genetic improvement. Rates of genetic gain in soybean have been estimated at about 17 to 22 kg/ha/year, but potentially can be improved.^1–3^

With the advancements in genotyping technologies and sequencing, an important increment in the number of delivered markers at low cost can be achieved, which can open opportunities to increase genetic gain in soybean. Genomic prediction (GP) is a technique that is widely used in breeding programs for cultivar development, and it aids the selection process by taking advantage of the use of molecular markers for estimating the performance of lines based on their genomic estimated breeding value. It is more effective than traditional phenotypic or pedigree-based selection, and it has the potential to increase genetic gain by threefolds^4^ compared with marker-assisted selection. Genomic prediction is a procedure that combines genotypic and phenotypic information to build prediction models and performs prediction on un-phenotyped lines using only their marker profiles. The technique was first introduced by Meuwissen et al.^5^ Since then, a lot of effort was devoted to model development in GP,^6–9^ implementation of GP,^10,11^ and model comparison.^12-14^

Another avenue to improve GP models is to optimize them according to the data available for prediction. Howard et al^14^ used response surface methodology to optimize GP models based on number of lines, number of markers, number of quantitative trait loci, degree of epistasis (gene-by-gene interaction), and degree of heritability (proportion of phenotypic variability explained by the genetic variability) in a simulated data set. There are studies that aim to improve GP models by optimizing the relationship between the training and the testing sets.^15^ Genomic prediction models can also be improved by optimizing the quality control (QC) of the genotypic data used for model development. Jarquín et al^16^ compared prediction models for a soybean population grown by the University of Nebraska—Lincoln Soybean Breeding Program with different degrees of missingness and minor allele frequency (MAF) in the genomic marker data. However, the focus of the study was not to evaluate a comprehensive set of factors considered in QC but to evaluate the genotype-by-sequencing genotyping technology in GP for soybean breeding.^16^

In this study, we evaluated GP accuracy based on QC of genomic data collected on soybean populations grown by the University of Nebraska—Lincoln Soybean Breeding Program. We varied the sets of markers to be included in the model by considering different percentages of missing values (PMMS; 27 levels) and different levels for MAF (12 levels). Training and testing sets for all these combinations (27 × 12 = 324) were conformed 200 times. The evaluation of this comprehensive set of combinations offered the opportunity to construct the response surface of the prediction accuracy (based on the percentage of missing genomic values and the MAF). As the genotyping by sequencing (GBS) technology is not perfect and a large number of missing values are delivered, 2 imputation methods were implemented to compare their effects on the predictive ability of the models (naïve imputation method [where the mean of the non-missing marker values are inserted for the missing markers] and the Random Forest (RF)-based imputation). In this context, several novelty methods have been developed for imputing missing data. Some of these consider haplotype phase information,^17^ others use information from higher density panels from reference individuals,^18^ or are based on classification and regression methods for unordered markers.^19^ A compressive review of the impacts on predictive ability of several imputation methods can be found in Rutkoski et al.^20^

In this article, first the phenotypic and genotypic marker data that were used for GP are introduced. Then, it is described how the QCs were implemented for the different factors and levels used for constructing the response surface of the prediction accuracy values for the 2 imputation methods. Briefly, the GP model that was implemented is also introduced. Finally, we discuss the response surface of the prediction accuracy values dependent on the level of missing marker values, the MAF, and the imputation technique (IT) used to create more comprehensive sets of genomic data, and some conclusions based on our findings are provided.

## Material and Methods

### Phenotypic and genotypic data

The predictions were conducted using phenotypic and genotypic data on 301 soybean lines grown by the University of Nebraska—Lincoln Breeding Program. These lines belong to 3 maturity groups (MG) [64, 213, and 24 lines from MGs I, II, and III, respectively] and were tested in 6 locations in Nebraska (Beemer [277], Phillips [301], Cotesfield [277], Mead [301], Lincoln [24], and Clay center [24]). Only in the Phillips and Mead locations were all the lines tested. A complete description of the distribution of the lines and the experimental design can be found in Jarquín et al.^16^ Phenotypes of 3 traits were considered in the analysis: grain yield (GY), days to maturity (DTM), and plant height (PH). The phenotypes were adjusted accounting for the location and block effects due to the experimental design. The genomic data, the genotyping procedure, and the GBS analysis are described in detail in Jarquín et al.^16^ Briefly, DNA isolation was performed using the Qiagen DNeasy Plant 96 kit, and the samples were analyzed in the Institute of Genomic Diversity at Cornell University. Then, the GBS analysis pipeline implemented in Tassel Version 3.0.156 was used to call the single-nucleotide polymorphisms (SNPs). After the SNP calling, the molecular marker information consisted of 216K SNP markers.

### GP model

The GP model used to evaluate prediction accuracy for the 3 traits (GY, PH, and DTM) was the genomic best linear unbiased prediction (G-BLUP) model including only additive effects. The model can be written as

$$y_{i}=\mu +g_{i}+\varepsilon_{i},$$

where *y_i_* is the phenotype of the *i*^th^ line (*i* = 1, …, *n*), μ is the overall mean, *g_i_* is the additive genetic value of the *i*^th^ line, and ε_*i*_ is the corresponding residual term.

Using matrix notation, the model can be written as **y** = **μ** + **g** + **ε**, where **g** = **Xb** with **X** being a *n *× *p* (*n* is the number of genotype and *p* is the number of markers) dimensional matrix of genotype scores. Considering that the marker effects associated with the *j*^th^ marker (i.e. *X_j_*; for *j* *=* 1, 2, …, *p*) are distributed as $N(0,\sigma_{b}^{2})$ and based on the assumptions of the multivariate normal distribution, the mean and co-variance of **g** (the vector of genetic effects) are the null vector **0** and $\mathrm{Cov}(g)=XX^{\prime }\sigma_{b}^{2}=G\sigma_{g}^{2}$ where $G=p^{-1}XX^{\prime }$ and $\sigma_{g}^{2}=p\sigma_{b}^{2}$. Summarizing the model, we can write $g∼N(0;G\sigma_{g}^{2})$ and $\varepsilon_{i}∼N(0;\sigma_{\varepsilon }^{2})$, where **G** is commonly referred to as the Genomic Relationship Matrix and its entries describe the genetic similarities among pair of lines.

The model was evaluated based on prediction accuracy, which was defined as the first moment Pearson correlation coefficient between the observed phenotypic value and the predicted genomic-enabled breeding value. The predictions were carried out using a tenfold cross-validation scheme, which was repeated 200 times. Then, the mean and the variance of the prediction accuracy values were calculated.

### QC of the genomic data

Quality control is a fundamental step in genomic data analysis and GP, and it might significantly influence the prediction accuracy. In our study, we focused on evaluating 3 of the most important factors in QC. These are done after the genomic data are translated to a numeric format. These 3 factors were the MAF, the percentage of missing marker scores (PMMS), and the IT used to complete the genotypic data. Minor allele frequency is the proportion of the second most common allele occurring in a population. Using genomic data, it is calculated as the frequency of the second most common allele among the genotyped lines. This value provides information about the proportion of common versus rare variants in the population. The procedure consists in discarding markers from the analysis with a MAF smaller than a given cut-off value. In genomic studies, different cut-off points have been adopted for discarding makers based on MAF. Thus, there is not a conventional value used in all species. For example, in maize^21^ and wheat,^22^ a MAF of 0.05 was used for GBS and an Infinitum SNP array, respectively, while^23^ considered a cut-off of 0.01 in wheat for GBS data. PMMS is the percentage of missing marker scores is the percentage of marker scores in the genomic data set that are missing. A large percentage of missing values would lead to inaccurate estimations of markers’ effects delivering biased and incorrect predictions. Thus, markers with a large PMMS should be avoided in the analysis. For this factor, these previous authors considered a similar cut-off criterion and discarded those markers with more than 20%, 15%, and 20%, respectively.

The purpose of the study was to evaluate prediction accuracy using genomic data where MAF and PMMS are varied under 2 ITs (naïve and RF). The response of the prediction accuracy under different combinations of the levels of MAF and PMMS was visualized by a response surface plot. The response surface was evaluated at all of the pairwise combinations of 12 levels of MAF and 27 levels of PMMS. The response surface was examined for highest peak, and 4 common MAF × PMMS combinations were evaluated. Also, the number of markers at each MAF × PMMS combinations were computed, and the values were added in the bottom part of the response surface plot using a scaled surface.

## Results and Discussion

In this study, the response surface of GP accuracy was created as a function of the combination of MAF and PMMS, and 2 ITs used for imputing missing values in the genomic data were compared. For the predictions, genomic and yield data from the University of Nebraska—Lincoln Soybean Breeding Program were used.

**Figure 1.**
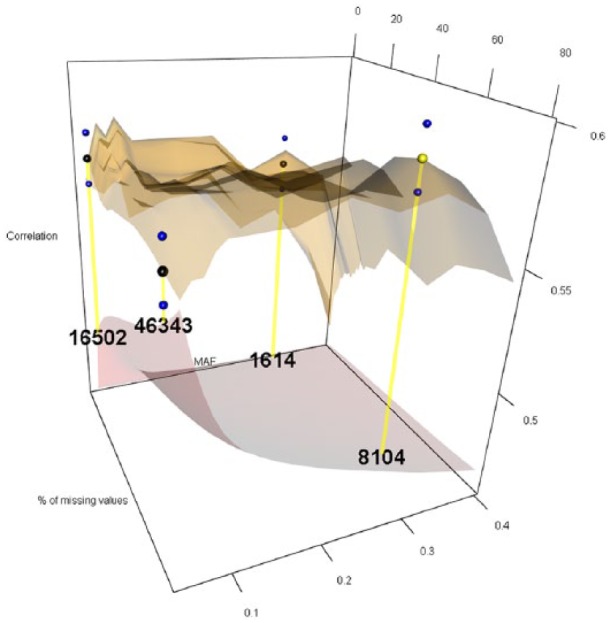
Response surface plot (top) of the prediction accuracy values as a function of MAF (0.05, 0.06, 0.07, 0.08, 0.09, 0.10, 0.15, 0.20, 0.25, 0.30, 0.35, and 0.40) and PMMS (1%, 2%, 3%, 4%, 5%, 6%, 7%, 8%, 9%, 10%, 11%, 12%, 13%, 14%, 15%, 16%, 17%, 18%, 19%, 20%, 25%, 30%, 40%, 50%, 60%, 70%, and 80%) for grain yield using the naïve imputation technique. The black and blue dots represent the mean prediction accuracy for 4 particular MAF × PMMS combinations ([0.05, 5%], [0.05, 70%], [0.3, 5%], and [0.3, 70%]) and the obtained standard deviations for 200 replicates of training-testing randomizations. The gray response surface (bottom) represents the number of markers that remains in the analysis after applying the quality controls (QCs) on marker data (MAF and PMMS). The numbers in the gray plot correspond to the actual number of markers that remained in the analysis for the 4 particular MAF × PMMS combinations. The yellow dot points at the combination that gave the highest correlation (0.586 and SD: 0.011). In this case, the yellow and black points coincided for the (0.3, 70%) combination with 8104 marker SNPs. MAF indicates minor allele frequency; PMMS, percentage of missing marker scores; SNP, single-nucleotide polymorphism.

**Figure 2.**
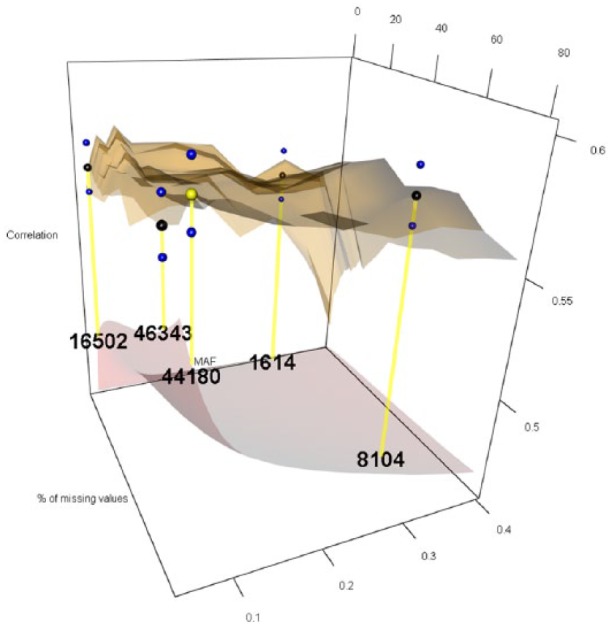
Response surface plot (top) of the prediction accuracy values as a function of MAF (0.05, 0.06, 0.07, 0.08, 0.09, 0.10, 0.15, 0.20, 0.25, 0.30, 0.35, and 0.40) and PMMS (1%, 2%, 3%, 4%, 5%, 6%, 7%, 8%, 9%, 10%, 11%, 12%, 13%, 14%, 15%, 16%, 17%, 18%, 19%, 20%, 25%, 30%, 40%, 50%, 60%, 70%, and 80%) for grain yield using the Random Forest imputation technique. The black and blue dots represent the mean prediction accuracy for 4 particular MAF × PMMS combinations ([0.05, 5%], [0.05, 70%], [0.3, 5%], and [0.3, 70%]) and the obtained standard deviations for 200 replicates of training-testing randomizations, respectively. The gray response surface (bottom) represents the number of markers that remains in the analysis after applying the quality controls (QCs) on marker data (MAF and PMMS). The numbers in the gray plot correspond to the actual number of markers that remained in the analysis for the 4 particular MAF × PMMS combinations. The yellow dot points at the combination (0.06, 80%) that gave the highest correlation (0.591, SD: 0.010) with 44 180 marker SNPs. MAF indicates minor allele frequency; PMMS, percentage of missing marker scores; SNP, single-nucleotide polymorphism.

**Figure 3.**
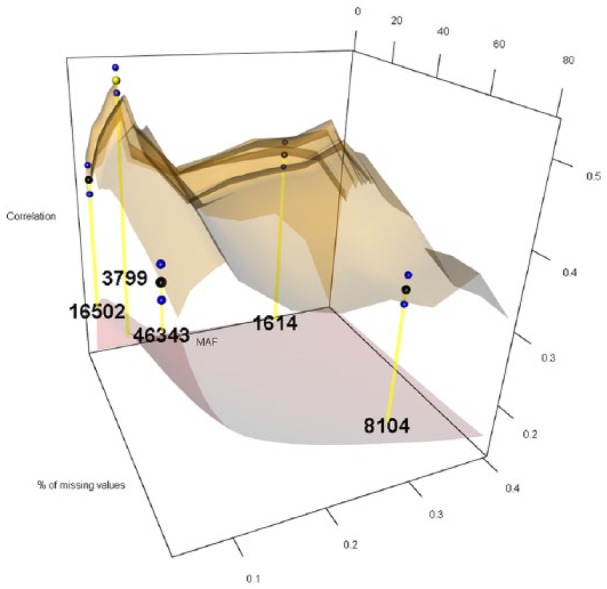
Response surface plot (top) of the prediction accuracy values as a function of MAF (0.05, 0.06, 0.07, 0.08, 0.09, 0.10, 0.15, 0.20, 0.25, 0.30, 0.35, and 0.40) and PMMS (1%, 2%, 3%, 4%, 5%, 6%, 7%, 8%, 9%, 10%, 11%, 12%, 13%, 14%, 15%, 16%, 17%, 18%, 19%, 20%, 25%, 30%, 40%, 50%, 60%, 70%, and 80%) for plant height using the naive imputation technique. The black and blue dots represent the mean prediction accuracy for 4 particular MAF × PMMS combinations ([0.05, 5%], [0.05, 70%], [0.3, 5%], and [0.3, 70%]) and the obtained standard deviations for 200 replicates of training-testing randomizations, respectively. The gray response surface (bottom) represents the number of markers that remains in the analysis after applying the quality controls (QCs) on marker data (MAF and PMMS). The numbers in the gray plot correspond to the actual number of markers that remained in the analysis for the 4 particular MAF × PMMS combinations. The yellow dot points at the combination (0.09, 2%) that gave the highest correlation (0.515, SD: 0.015) with 3799 marker SNPs. MAF indicates minor allele frequency; PMMS, percentage of missing marker scores; SNP, single-nucleotide polymorphism.

**Figure 4.**
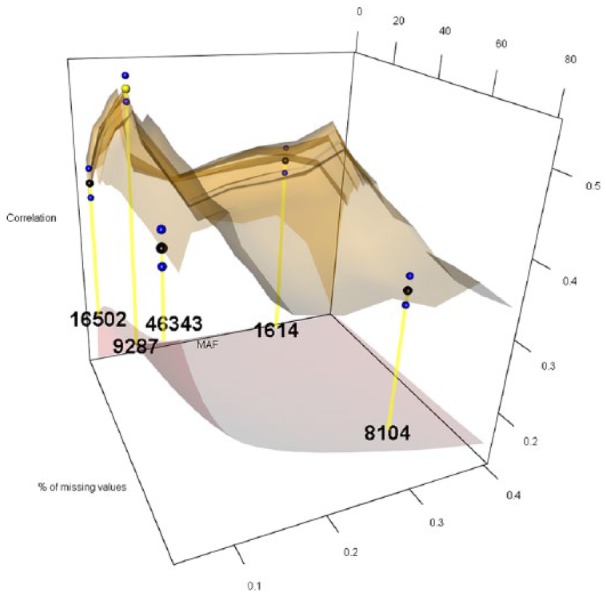
Response surface plot (top) of the prediction accuracy values as a function of MAF (0.05, 0.06, 0.07, 0.08, 0.09, 0.10, 0.15, 0.20, 0.25, 0.30, 0.35, and 0.40) and PMMS (1%, 2%, 3%, 4%, 5%, 6%, 7%, 8%, 9%, 10%, 11%, 12%, 13%, 14%, 15%, 16%, 17%, 18%, 19%, 20%, 25%, 30%, 40%, 50%, 60%, 70%, and 80%) for plant height using the Random Forest imputation technique. The black and blue dots represent the mean prediction accuracy for 4 particular MAF × PMMS combinations ([0.05, 5%], [0.05, 70%], [0.3, 5%], and [0.3, 70%]) and the obtained standard deviations for 200 replicates of training-testing randomizations. The gray response surface (bottom) represents the number of markers that remains in the analysis after applying the quality controls (QCs) on marker data (MAF and PMMS). The numbers in the gray plot correspond to the actual number of markers that remained in the analysis for the 4 particular MAF × PMMS combinations. The yellow dot points at the combination (0.09, 11%) that gave the highest correlation (0.524, SD: 0.015) with 9287 marker SNPs. MAF indicates minor allele frequency; PMMS, percentage of missing marker scores; SNP, single-nucleotide polymorphism.

**Figure 5.**
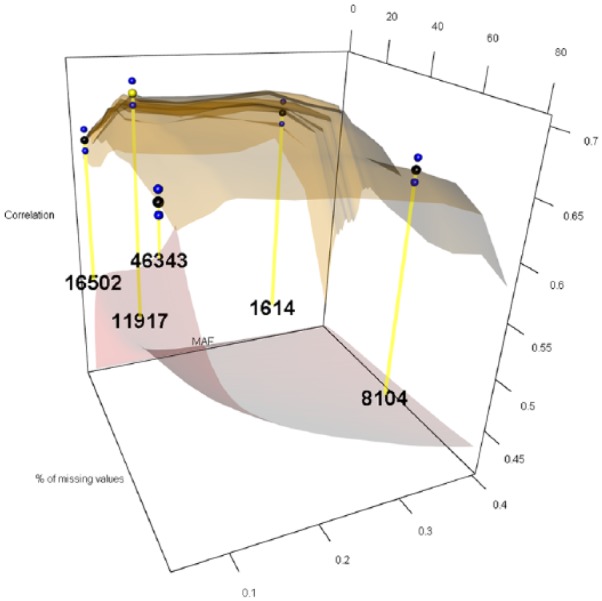
Response surface plot (top) of the prediction accuracy values as a function of MAF (0.05, 0.06, 0.07, 0.08, 0.09, 0.10, 0.15, 0.20, 0.25, 0.30, 0.35, and 0.40) and PMMS (1%, 2%, 3%, 4%, 5%, 6%, 7%, 8%, 9%, 10%, 11%, 12%, 13%, 14%, 15%, 16%, 17%, 18%, 19%, 20%, 25%, 30%, 40%, 50%, 60%, 70%, and 80%) for days to maturity using the naive imputation technique. The black and blue dots represent the mean prediction accuracy for 4 particular MAF × PMMS combinations ([0.05, 5%], [0.05, 70%], [0.3, 5%], and [0.3, 70%]) and the obtained standard deviations for 200 replicates of training-testing randomizations. The gray response surface (bottom) represents the number of markers that remains in the analysis after applying the quality controls (QCs) on marker data (MAF and PMMS). The numbers in the gray plot correspond to the actual number of markers that remained in the analysis for the 4 particular MAF × PMMS combinations. The yellow dot points at the combination (0.09, 20%) that gave the highest correlation (0.691, SD: 0.009) with 11 917 marker SNPs. MAF indicates minor allele frequency; PMMS, percentage of missing marker scores; SNP, single-nucleotide polymorphism.

**Figure 6.**
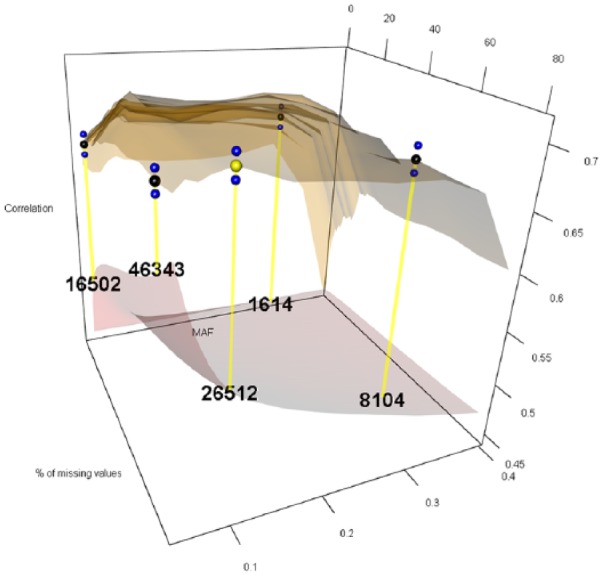
Response surface plot (top) of the prediction accuracy values as a function of MAF (0.05, 0.06, 0.07, 0.08, 0.09, 0.10, 0.15, 0.20, 0.25, 0.30, 0.35, and 0.40) and PMMS (1%, 2%, 3%, 4%, 5%, 6%, 7%, 8%, 9%, 10%, 11%, 12%, 13%, 14%, 15%, 16%, 17%, 18%, 19%, 20%, 25%, 30%, 40%, 50%, 60%, 70%, and 80%) for days to maturity using the Random Forest imputation technique. The black and blue dots represent the mean prediction accuracy for 4 particular MAF × PMMS combinations ([0.05, 5%], [0.05, 70%], [0.3, 5%], and [0.3, 70%]) and the obtained standard deviations for 200 replicates of training-testing randomizations, respectively. The gray response surface (bottom) represents the number of markers that remains in the analysis after applying the quality controls (QCs) on marker data (MAF and PMMS). The numbers in the gray plot correspond to the actual number of markers that remained in the analysis for the 4 particular MAF × PMMS combinations. The yellow dot points at the combination (0.1, 80%) that gave the highest correlation (0.704, SD: 0.009) with 26 512 marker SNPs. MAF indicates minor allele frequency; PMMS, percentage of missing marker scores; SNP, single-nucleotide polymorphism.

The 12 levels considered for MAF were 0.05, 0.06, 0.07, 0.08, 0.09, 0.10, 0.15, 0.20, 0.25, 0.30, 0.35, and 0.40, while the 27 levels for PMMS were 1%, 2%, 3%, 4%, 5%, 6%, 7%, 8%, 9%, 10%, 11%, 12%, 13%, 14%, 15%, 16%, 17%, 18%, 19%, 20%, 25%, 30%, 40%, 50%, 60%, 70%, and 80%. For imputing the marker scores, we compared the naïve and RF ITs. Figures 1-6 represent the response surface plots of the GP accuracy values depending on the MAF and PMMS. Figures 1 and 2 are for GY, Figures 3 and 4 are for PH, and Figures 5 and 6 are for DTM. For Figures 1, 3, and 5, the naïve IT was implemented, and for Figures 2, 4, and 6, the RF technique was implemented. Table 1 shows results for 4 MAF × PMMS combinations that were examined for all of the 6 figures (trait × IT).

**Table 1.** The levels of MAF, PMMS, and corresponding number of markers for the 4 examined cases and those combinations that gave the highest predictive ability in the response surface plots in terms of average prediction accuracy, and corresponding standard errors for grain yield (GY), plant height (PH), and days to maturity (DM) for the 2 imputation methods.

MAF	PMMS	No. of SNPs	GY-Naive	GY-RF	PH-Naive	PH-RF	DM-Naive	DM-RF
0.05	5	16 502	0.566 (0.010)	0.565 (0.010)	0.396 (0.019)	0.402 (0.019)	0.644 (0.009)	0.647 (0.009)
0.05	70	46 343	0.560 (0.010)	0.577 (0.010)	0.402 (0.018)	0.445 (0.018)	0.662 (0.008)	0.687 (0.008)
0.3	5	1614	0.557 (0.012)	0.555 (0.011)	0.405 (0.018)	0.408 (0.018)	0.657 (0.010)	0.659 (0.010)
0.3	70	8104	0.586 (0.011)	0.577 (0.011)	0.347 (0.018)	0.356 (0.018)	0.666 (0.009)	0.684 (0.010)
			**0.586 (0.011)**	**0.591 (0.010)**	**0.515 (0.015)**	**0.524 (0.015)**	**0.691 (0.009)**	**0.704 (0.009)**
			**(0.3, 70%, 8104)**	**(0.06, 80%, 44 180)**	**(0.09, 2%, 3799)**	**(0.09, 11%, 9287)**	**(0.09, 20%, 11 917)**	**(0.10, 80%, 26 512)**

Abbreviations: MAF, minor allele frequency; PMMS, percentage of missing marker scores; RF, Random Forest; SNP, single-nucleotide polymorphism.

Besides the 4 common coordinates, the highest peak is also shown on the response surface plots. In only one case, the highest peak was also 1 of the 4 commonly evaluated coordinates. The black dots represent the mean prediction accuracy values for the 4 common coordinates, and the yellow dots are the mean prediction accuracy values for the highest peak. The blue dots show the standard deviation of the prediction accuracy using the 200 replicates for a 10-fold cross-validation design. The numerical values within the plots represent the number of markers used at those coordinates (combinations).

For GY under the naïve IT (Table 1 and Figure 1), the highest correlation (0.586) was obtained when markers with a MAF of at least 0.3 and less than 70% of missing values remained in the analysis delivering a total of 8140 SNPs. In this case, the highest value coincided with 1 of the common QCs for a slight improvement compared with the other 3 cases. Under the RF imputation, the highest correlation (Table 1 and Figure 2) was slightly higher (0.591) compared with the naïve imputation. This value was obtained when a MAF of 0.06 and PMMS equal to 80% were used as QCs. Thus, for reaching same levels of predictive ability, a larger number of markers were necessary (44 180) under RF IT. Thus, no significant differences were found for the highest correlation obtained between the different IT; however, these values were obtained using different QCs resulting in different number of markers. In this case, the naïve imputation needed less than 20% of the markers that were necessary for the RF to reach comparable results. The same statement applies for the 4 different QCs’ combinations within each IT but not along the complete surface response. Comparing Figures 1 and 2, we observe a flatter surface response (especially in the corners of the surface) when the RF imputation was implemented indicating a small improvement in predictive ability along the complete surface by using this imputation method.

Results for PH (Table 1 and Figures 3 and 4) showed different response surface patterns than for GY, which was expected due to the different genetic architecture of this trait. As shown by Fang et al,^24^ this trait is controlled by a very few loci. Here, the RF imputation gave the highest predictive ability (0.524) using approximately 2.5 times more markers (9287) than the naïve imputation (3799), which produced a mean correlation of 0.515. Similar to the previous case, the response surface obtained by the RF seemed a little flatter than the surface obtained by the other imputation method. Despite the imputation method, there were sizable improvements in predictive ability with respect to conventional QCs. These improvements ranged between 27% and 48% for naïve imputation, while for RF, it was between 17% and 47%. Hence, a clear advantage was shown by considering other than conventionally used QCs.

Days to maturity showed a slight improvement in predictive ability by using the RF imputation compared with the naïve method. Similarly to the other 2 traits, the response surface was flatter (especially in the corners) using the RF technique. This IT gave the highest correlation (0.704) using 2.2 times more markers (26 512) than what was necessary with the naïve method (11 917), which delivered a mean correlation of 0.691. For this trait, the improvements with respect to conventional QCs ranged between 2% and 7% for both imputation methods. Thus, no significant improvements were observed considering other values than the commonly used QCs.

In this study, we showed that the improvements in predictive ability are affected by (1) the genetic architecture of the trait and (2) the imputation method as well. The highest correlations were found considering different combinations of MAF and PMMS, which also varied the number of markers necessary for the analysis. In general, RF produced flatter response surfaces showing a slight advantage by using this imputation method. Also, this method needed between 2 and 5 times more markers than the naïve imputation for producing comparable results. Finally, we saw sizable, moderate, and null improvements in predictive ability for PH, GY, and DTM, respectively, by considering QCs other than those that are commonly used.
